# Acetabular Debonding: An Investigation of Porous Coating Delamination in Hip Resurfacing Arthroplasty

**DOI:** 10.1155/2018/5282167

**Published:** 2018-11-01

**Authors:** Eric Robinson, Dani Gaillard-Campbell, Thomas P. Gross

**Affiliations:** Midlands Orthopaedics & Neurosurgery, 1910 Blanding Street, Columbia, SC 29201, USA

## Abstract

**Background:**

To date, there have been no published investigations on the cause of acetabular debonding, a rare failure phenomenon in metal-on-metal hip resurfacing where the acetabular porous coating delaminates from the implant while remaining well fixed to the pelvic bone.

**Purposes:**

This study aims to summarize the current understanding of acetabular debonding and to investigate the discrepancy in rate of debonding between two implant systems.

**Patients and Methods:**

To elucidate potential causes of debonding, we retrospectively analyzed a single-surgeon cohort of 839 hip resurfacing cases. Specifically, we compared rate of debonding and manufacturing processes between two implant systems.

**Results:**

Group 1 experienced significantly more cases of debonding than Group 2 cases (4.0% versus 0.0%, p value<0.0001). Implant manufacturing processes differed in surface coating, heat treatment, postmanufacturing treatment, and apex thickness. Debonded implants were more likely to have missed RAIL guidelines (p=0.04).

**Conclusions:**

We identified implant system, postoperative time, and acetabular component placement as variables contributing to rate of debonding. We recommend minimizing acetabular inclination angle according to RAIL guidelines. Further, we evaluated manufacturing differences between the two implant systems but did not have access to proprietary data to identify the cause of debonding. Both implants met ASTM standards, yet only the Group 1 implant debonded. This suggests the second implant had greater fatigue shear strength. Because the Group 2 implant achieved a more durable interface that did not debond, we suggest the ASTM F1160 standard for fatigue shear strength be increased to that achieved by its manufacturer.

**Level of Evidence II:**

A retrospective evaluation of prospectively collected data.

## 1. Introduction

After early enthusiasm in the 2000s [1, 2], hip resurfacing arthroplasty (HRA) fell into disfavor due to a high incidence of metallosis with poorly designed implants and at outlier centers [3]. HRA is more technically demanding than total hip arthroplasty (THA) and necessitates a separate, steeper learning curve. Available data suggest HRA performed by experienced resurfacing surgeons offers greater functional capacity and improved durability over THA [4–6]. Other advantages include superior hip stability [7–9], greater bone preservation leading to less-challenging revision surgeries [10–12], and lower 10-year all-cause mortality in matched patient populations when compared to THA [13].

To further improve the success and performance of HRA, research groups should evaluate failure modes of resurfacing systems and devise appropriate solutions. In 2012, the FDA presented a memorandum discussing safety and efficacy of metal-on-metal (MoM) hip implant systems [14]. This report identified common failure modes of MoM systems, but despite the substantial level of detail, the memorandum failed to mention a rare and poorly understood failure mode in MoM HRA implant systems, debonding of the porous titanium (Ti) coating from the cobalt (Co) chrome acetabular component.

Debonding occurs when shear forces applied at the bone-implant interface are too high, causing patches of porous coating to shear from the cup upon loading. Acetabular debonding has been investigated in a few, small case studies [15–17], but to date, no studies have been published reporting rates of debonding in a single, large cohort. Debonding presents an unusual failure mode that may illuminate flaws in implant design or manufacturing processes. The current paper presents a retrospective analysis of a single-surgeon database with over 5000 HRA cases. We compared the rate of debonding as a unique failure between the two different HRA implant systems used in our practice: the Corin Cormet 2000 and Biomet M2a Magnum-ReCap™ systems. The purpose of this paper was twofold: (1) to summarize the current literature and present a hypothetical mechanism of debonding failure and (2) to evaluate any discrepancy in rate of debonding between the two implant systems by investigating differences in the implant manufacturing and testing processes.

## 2. Methods

### 2.1. Patients and Follow-Up Method

The senior author (TPG) maintains a prospective database (OrthoVault, Columbia, SC) of more than 5000 HRA procedures. This surgeon has performed HRA since 1991, and therefore, none of these cases are from his initial learning curve. The current study analyzes 839 consecutive procedures between January 2001 and December 2007, with a minimum 10-year follow-up. Approvals for this study and report were obtained from the Institutional Review Board of Lifepoint Health Providence Hospitals in Columbia, South Carolina. In this study, we compare two similar implants with respect to a single failure mechanism: late acetabular component loosening. The first cohort (Group 1) comprised 329 Corin Cormet devices implanted between 2001 and 2004. The second group (Group 2) consisted of 510 Biomet Magnum™ cases performed between 2004 and 2007. The two groups were demographically similar, although Group 2 was slightly older (p value=0.01) with a higher preoperative clinical score (p value=0.0024), on average (Table 1). Group 2 had more patients with posttrauma HRA (2.7% versus 0.3%, p=0.009). Further, Group 1 had a higher proportion of patients with osteonecrosis (11.2% versus 4.1%,* p* <0.0001), although no HRA cases with a diagnosis of osteonecrosis reported acetabular debonding.

### 2.2. Implant Systems

The senior author used these two implant systems in a consecutive fashion. Between March 2001 and December 2004, the hybrid cemented Corin Cormet 2000 system (Corin, Cirencester, UK) was employed as a part of a multicenter United States FDA trial. Between December 2004 and December 2007, Biomet devices comprised a cemented ReCap™ femoral component and an uncemented Magnum™ acetabular component (Biomet, Warsaw, Indiana) and were used in an off-label fashion for total hip resurfacing.

**Figure 1:**
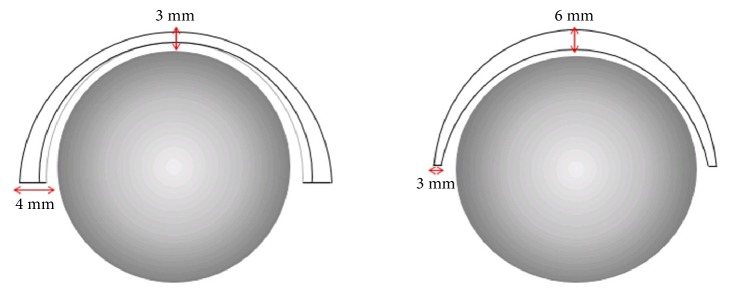
Implant design sketches. Corin (left) and Biomet (right) cup dimensions shown, with the gray sphere representing the femoral head.

Both groups employed similar hybrid MoM HRA implants, and each cohort had a minimum follow-up of 10 years. Similarities between the two acetabular components (Table 2) include a cast, high-carbon (>0.2%) CoCr substrate shot blasted with alumina grit and then coated with plasma-sprayed, unalloyed Ti. The coverage arcs are similar between these two implants, but the Group 2 implant arc is 4° less for each implant size on average. There are also several, notable differences in the manufacturing processes of these two devices. The Group 1 implant [24] is double heat-treated, and an additional layer of hydroxyapatite (HA) is plasma sprayed onto the Ti layer. Two sets of relatively thick antirotation fins at the equator imbed into the ischium and pubis during impaction. The Group 2 implant system is not heat-treated and does not have an HA layer. There are four pairs of smaller, thinner fins evenly spaced around the circumference of the implant. Implant dimensions are roughly sketched in Figure 1.

Several details, including those of the porous coating processes, are proprietary and not known to us. Average bead size was only available for the Cormet device [25]. Pore size range, but not mean pore size, was available for the Biomet Magnum [19]. We were unable to find time to fixation for either device.

### 2.3. Procedure

All procedures were performed using a posterior approach as described previously [26]. Before 2009, acetabular components were positioned with an acetabular inclination angle (AIA) under 55°, based on research by De Smet [27].

### 2.4. Clinical and Radiographic Analysis

Office or remote follow-up was requested at 6 weeks, 1 and 2 years, and every other year thereafter. A clinical questionnaire, radiographs, and a physical examination testing range-of-motion and strength were performed at each visit. After 1 year, physical examinations were no longer done routinely on remote follow-ups. The OrthoVault database (Midlands Orthopaedics & Neurosurgery, Columbia, South Carolina) supported our collection and analysis of demographic, clinical, and radiographic data for all patients.

Patient questionnaires facilitated the collection of information necessary for calculating the following clinical scores: Harris hip score (HHS) [28], University of California, Los Angeles (UCLA) activity score [29], and visual analogue scale (VAS) pain score for normal and worst days [30]. HHS determines clinical outcome; UCLA activity scores measure activity level on a scale from 1 to 10, for which 10 represented the highest level of activity; VAS pain scores rate the level of pain from 0 to 10, with zero representing no pain and 10 representing maximum levels of debilitating pain.

Both supine and standing anterior-posterior pelvis and lateral radiographs are taken and analyzed for component position, shifting, and radiolucencies by the senior author at each follow-up interval and upon request. We determine the acetabular inclination angle (AIA) on a standing pelvis radiograph by measuring the angle formed by two straight lines: one parallel to flat face of the acetabular component and the other tangential to the precipice of both ischial tuberosities. We measured all radiographs using InteleViewer (InteleRAD, Chicago, IL, USA).

### 2.5. Statistical Methods

The significance level *α* was defined as 0.05 for all statistical analyses in this study. A paired, two-tailed Student's T-test was used to calculate the significant difference between average preoperative and postoperative numerical outcomes within and between study groups. When comparing two population proportions, a two-sample Z-test was used. Kaplan-Meier survivorship curves were plotted to evaluate implant survival among different groups. Log-rank and Wilcoxon tests were performed to calculate significant differences between survivorship curves. Curves and survivorship statistical tests were generated using XLSTAT (New York, NY).

## 3. Results

**Figure 2:**
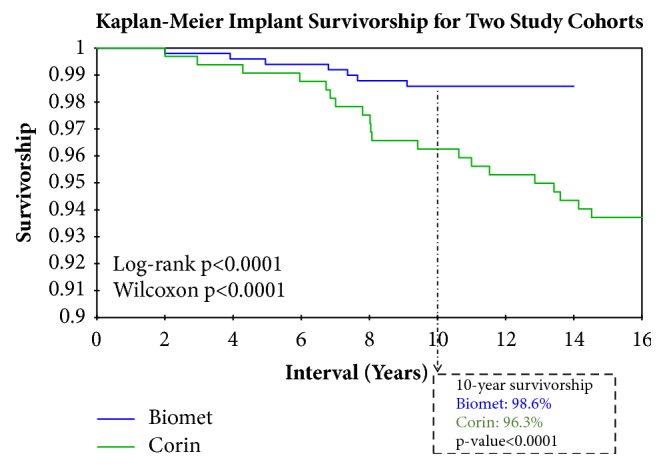
Kaplan-Meier implant survivorship for two study cohorts. Kaplan-Meier implant survivorship analysis using late acetabular failure as an endpoint. All cases in both groups have a minimum of 10 years' follow-up. At 10 years, survivorship is 97.2% for Biomet and 96.0% for Corin (p=0.0008). Late acetabular failure is defined as failure of acetabular component >2 years. Results of the log-rank test (p value <0.0001) and Wilcoxon test (p value <0.0001) show significant difference in the occurrence of late acetabular failures between the two implant groups.

In Group 1, there was a significantly higher rate of late acetabular component loosening, as well as loosening categorized as debonding; cup loosening characterized as debonding is confirmed intraoperatively. Both groups had a minimum of 10 years' follow-up, at which point Kaplan-Meier (KM) implant survivorship was 96.0% and 97.2%, respectively (p=0.0008) (Figure 2). With debonding as an endpoint, 10-year Group 1 implant survivorship was 97.8% and Group 2 implant survivorship was 100% (p<0.0001).

Table 3 lists the incidence of acetabular failure modes; definitions and descriptions of each failure mode are detailed in Table 4. Debonding only presented in Group 1, occurring at a mean of 9.7 years (range 4 to 14 years). The surgeon identified debonding intraoperatively at revision surgery, and this was confirmed by the surgical assistance and implant company representative; the implant coating appeared delaminated from the implant bulk material and well fixed in the pelvic bone. The surgical team only recorded where debonding occurred in three cases (23% of debonding cases). Of these, delamination was reported to have occurred on the outer edges of the cup. Amount of delamination ranged from 33 to 90% of the acetabular porous coating. Other acetabular failure modes, such as failure of ingrowth, AWRF, and late loosening with intact porous coating, were not different between the two groups (p value=0.2, p value=0.5, and p value=0.3, respectively). One Group 1 patient had revision surgery elsewhere, and therefore we categorized their failure as “unknown” loosening.

**Figure 3:**
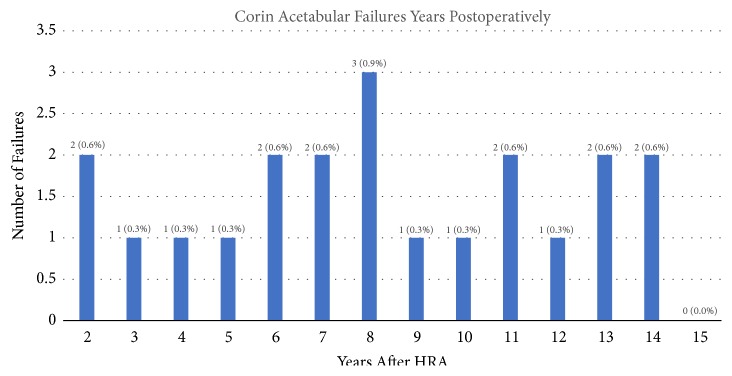
Corin late acetabular failure distribution over postoperative time. Figure 3 presents a distribution of Corin acetabular failures over postoperative time. Callouts show number of failures and percentage of the entire cohort (371 cases). Two cases failed before 2 years postoperatively (0.5% of cases) and are considered failure of ingrowth.

Figure 3 presents the number of late acetabular failure cases for Group 1 implant systems, dependent on time since surgery. Two Group 1 acetabular failures occurred before 2 years (0.5% of total cases) with all other acetabular failures occurring thereafter (5.1%). Failures occurred at a mean of 8.5 years. There were no differences between the time since surgery and rate of late failure. There was no statistical difference between date of HRA surgery and rate of late failure, although we observed most instances of Group 1 debonding were from cases performed in 2002. Manufacturers often use outside suppliers to perform porous coating. Unfortunately, the manufacturer would not provide us with vendor information for failed implants. Therefore, we investigated failure rate of implants from specific time periods; we could not establish any correlation. Group 2 implants failed late at 7 and 8 years. Clinical data (Table 5) indicate a higher postoperative HHS and UCLA activity score for Group 2 (HHS p value<0.0001, UCLA p value<0.0001), as well as lower VAS pain scores on regular and worst days (regular p value<0.0001, worst p value=0.0315). Radiographic data indicate a lower instance of radiolucency (p value<0.005) in Group 2 (Table 5). No debonding cases presented radiolucency. Nine debonding cases (69.2% total debonding cases) presented AIA over the relative acetabular inclination limit (RAIL) [32] on their earliest postoperative radiographs, as compared to 94 or 227 measured radiographs (41.4%) from Group 1 (p=0.04) (Table 6).

## 4. Discussion

We investigated a poorly understood failure phenomenon in which the porous coating debonds from the acetabular component. Several centers have published case studies on debonding [15–17]. However, our group is the first to analyze a large clinical database and report rate of acetabular component debonding in two implant systems.

We prospectively recorded details of all complications and failures in our database. For this study, we considered only acetabular failure modes, which include late loosening (>2 years), early loosening (<2 years), early cup shift (<2 years), and AWRF (Table 4). Results of the log-rank test (p value=0.0001) and Wilcoxon test (p value=0.0001) revealed implant choice as a significant factor in late acetabular failure, with 13 cases of Group 1 debonding out of 371 total surgeries (3.5%) (Figure 2). Group 2 presented 2 instances of late acetabular failure out of 728 surgeries (0.3%); during revision surgery, the surgeon (TPG) did not classify either of these complications as debonding.

Herein, we retrospectively analyzed clinical data routinely collected as part of standard care. We did not perform retrieval analyses on delaminated implants, although it would be beneficial in understanding where and how extensively debonding occurs. Debonding was confirmed intraoperatively during revision surgery by three professionals familiar with the surgery and product. All debonding patients reported abrupt onset of pain and presented a sudden cup shift on recent X-rays. Most recent metal ion levels prior to loosening were within optimal range, and the mean did not vary significantly from nonloosened cases (4.2 ± 2.7 *μ*g/L, and 2.6 ± 3.7 *μ*g/L, respectively; p value=0.1107). However, moderate metallosis was present intraoperatively in cases revised by the primary surgeon. All cases of debonding presented after 4 years postoperatively. Debonded cups met RAIL in only 30.8% of cases, as compared to 58.6% of all Group 1 cups (p=0.04). However, there was no difference in average AIA or number of cases above the RAIL between Group 1 and Group 2. Thus, cup position may contribute, but is not the sole cause, of debonding. Age, BMI, sex, diagnosis, and postoperative activity did not influence risk of debonding. We identified implant system, postoperative time, and AIA as factors that contribute to risk of debonding.

The available literature comprises three case studies on cup debonding in HRA [15–17] and three in THA [33–35]. These case studies suggest that debonding occurs when shear forces applied at the bone-implant interface are too high, causing patches of porous coating to shear from the cup upon loading. Delport* et al.* [15] found the surface coating of the debonded implant well fixed into the pelvis bone at revision surgery and reported signs of metallosis, although they did not publish metal ion values. Our group primarily performs HRA, and we have thus not encountered debonding in THA. However, we know four case studies on delamination [33–35]. Each group noted significant osteolysis and metallosis in their debonding cases. Amount of porous coating debonding ranged from 60 to 100% of the cup surface.

Manley* et al.* [36] found that the fatigue shearing strength of bead-sintered coating-substrate interfaces was similar to or less than that between coating and bone. An* in vivo* study found shearing strength at the bone-implant interface was between 4.25 and 7.81 MPa for plasma-sprayed, porous Ti coatings [31]. An* in vitro *analysis [24] of Ti porous coat-substrate interfaces found static shear strength to be greater than 20 MPa, on average; this same report showed the coating can withstand fatigue shear forces of 10 MPa for at least 10^7^ cycles, per the ASTM F1160 standard [14].

Corin reported static shear strength for the surface-substrate interface of the Cormet cup as 20.9 MPa in a batch of 5, but the standard deviation was 4.1 MPa [14]. Corin also completed shear fatigue strength testing on six samples for 10 million cycles at 10 MPa with no failures. Hot isostatic pressing (HIP) treatment of the Group 1 component may influence the bonding between the porous coat and substrate since HIP removes carbides. HIP may introduce residual stresses into the two materials, as the modulus of elasticity of the porous coat is one-third that of the substrate; however, it has been shown that HIP can increase the strength of adhesion between coat and substrate if done after the PVD process [37, 38].

Although we evaluated differences in implant design and manufacturing processes, we cannot be sure which design features caused Group 1 implants to debond significantly more than Group 2 cups. As described, Corin uses double heat treatment, HIP, and solution annealing, while Biomet does not. A study by Daniel* et al.* concluded that implants with double heat treatment present more wear and osteolysis [39]. With more information on the manufacturing process, we could know whether Corin's HIP introduces residual stresses into the implant, depending on which stage it is performed [37, 38]. Corin uses HA in their acetabular component coating, while Biomet does not; this may contribute to greater bone ingrowth and a stronger lock between bone and the implant surface [40]. In some accounts [21], sphericity between the components differs significantly. Lastly, the Biomet acetabular component has a thicker cup apex at 6 mm compared to Corin cups at 4 mm. Increased apex thickness prevents deformation during impaction, and thus, the Group 2 device may accumulate less damage intraoperatively [41]. We did not record the location of implant debonding in most cases, so we lack the information to identify a consistent pattern. However, of the cases that we did record details of debonding, we identified that delamination usually occurred on the outer edges of the cup and that 33 to 90% of the porous coating had detached from the implant and remained well fixed in the bone.

We hypothesize that debonding occurs at a higher rate in implants with a lower fatigue shear strength, and therefore, the ASTM F1160 testing standard for fatigue shear strength needs to be increased. Microfractures accumulate during daily and strenuous activity while osteocytes remodel bone and repair these microfractures [42]. However, the torsional strain and fatigue shearing stress transmit to the implant, which has no inherent repair capabilities. Thus, despite the greater shear strength at the substrate-coating interface, the bone-coating interface does not accumulate fatigue strain [43, 44]. Inelastic mismatch strain between the surface film and bulk material may also contribute to debonding of the coating [1, 40].

A primary limitation of this study stems from using these resurfacing devices in a consecutive fashion. Group 1 has a follow-up of 13 to 15 years, while the Group 2 is 10 to 13 years out from surgery. However, most acetabular failures occur before 10 years postoperatively (Figure 3). Additionally, to minimize this follow-up bias, we provide an additional comparison of all late loosening between the two groups that occurred before 10 years (2.2% of Group 1 cases, 0.3% of Group 2 cases, p value=0.002). Secondly, many details of the implant manufacturing processes are proprietary, and therefore another limitation is our lack of enough detailed information to identify the exact cause of debonding. Another limitation is our classification of debonding as a binary “yes or no”; detailing the mechanism or extent of debonding will require a future retrieval analysis study. However, the primary purpose of this study was to identify whether rate of debonding varied between implant brands and if there were any differences in the manufacturing processes. Lastly, the Corin group had fewer cases with posttraumatic HRA and was more diagnosed with osteonecrosis. However, no cases with osteonecrosis or posttraumatic HRA debonded.

## 5. Conclusion

This paper presents a retrospective analysis of a single-surgeon cohort, an implant manufacturer comparison, and a literature review of acetabular debonding, a rare and poorly understood failure mode. In debonding, bone ingrows into the implant initially, but late loosening occurs when the plasma porous coat on the outer face of the acetabular cup becomes detached from the bulk implant and remains fixed to the pelvic bone. Acetabular component position seems to have some influence; we recommend surgeons align acetabular cups according to the RAIL guidelines. Furthermore, the Group 1 acetabular cup failed from debonding at a rate of 3.5% while the Group 2 cup did not delaminate. We identified postoperative time, AIA, and implant type as risk factors for debonding. The differences in manufacturing processes and design (Table 2) for the Group 1 implant likely contribute to its high rate of debonding. According to the current available literature, debonding of the porous coat occurs due to accumulation of microfractures at the coating-substrate interface. Therefore, we believe that a fatigue failure test may be more relevant to this failure mode than a static test. We suggest that the ASTM F1160 standard for fatigue shear strength be increased to minimize the chance that implant coatings will debond even after years of vigorous activity.

## Figures and Tables

**Table 1:** Demographic information.

Demographic Information			
	Group 1	Group 2	

Variable	Corin	Biomet	P-value

Number of Cases	371	728	--

Age (years)	53.8 ± 9.3	55.1 ± 8.2	0.0177*∗*

BMI	28.2 ± 5.2	27.9 ± 4.7	0.3371

Female gender (%)	32%	29.1%	0.3125

Preoperative Harris Hip Score	43.4 ± 10.3	45.6 ± 11.8	0.0024*∗*

*Diagnosis (%)*			

Osteoarthritis	74.1%	81.6%	0.00398*∗*

Osteonecrosis	11.9%	4.3%	0.0001*∗*

Dysplasia	7.5%	8.7%	0.5287

Legg Perthes	0.8%	1.7%	0.2585

Post Trauma	0.3%	0.3%	0.9840

Rheumatoid Arthritis	1.1%	2.3%	0.1499

Slipped Capital Epiphysis	0.5%	1.0%	0.4654

Other	3.8%	0.3%	0.0001*∗*

*∗* represents significance.

**Table 2:** Comparison of acetabular component design and manufacturing processes.

	**Corin Cormet 2000**	**Biomet M2a-Magnum**
Acetabular Diameter Difference (Outer-Inner Diameter)	6-8 mm [18]	6 mm [19]

Bulk alloy	CoCrMo [18, 20]	CoCrMo [19]

Carbon content	“High” [4, 5], 0.2-.35% [18]	"High" [21], 0.23-.28% [19]

***Surface coating*** **∗**	Dual plasma spray (Ti+ HA) [18]	Plasma spray (Ti) [19]

***Heat treatment*** **∗**	Yes [18]	No [19]

***Manufacturing process*** **∗**	Cast [21] with hot isostatic pressing and solution annealing [18]	As cast [21]

Roughening of cast implant	Honed with a shotblast of alumina grit [22]	Honed with a shotblast of alumina grit [19]

Sphericity	3.8 *µ*m [21], <10 *µ*m [18]	1.9 *μ*m [21], 5 *μ*m [19]

Surface roughness	0.030 um [21]	0.031 um [21]

Equator/peripheral thickness	4 mm [23], 5.6 mm [21], 4.2 mm (our measurement)	3 mm [19], 3.4 mm [21], 3 mm (our measurement)

***Apex thickness*** **∗**	3 mm [23]	6 mm [19]

Coverage arc for implants with bearing size 40-56mm	160-166^0^ [20]	156-162^0^ [19]

Radial clearance	97.67 *µ*m (Medium) [21], 75-200 *µ*m [22]	120.93 *µ*m (High) [21], 75-150 *µ*m [19]

*∗*Bold with asterisks represents difference.

**Table 3:** Acetabular failures by category.

**Type**	**Corin**	**Biomet**	**P-value**
# Cases	371	728	--

*(1) Late Loosening*	15 (4.0%)	2 (0.3%)	<0.0001*∗*

Debonding	13 (3.5%)	0 (0.0%)	<0.0001*∗*

Intact Coating	0 (0.0%)	2 (0.3%)	0.3125

Unknown	1 (0.3%)	0 (0.0%)	0.1615

*(2) Early Loosening*	2 (0.5%)	8 (1.1%)	0.3576

*(3) AWRF*	4 (1.1%)	4 (0.5%)	0.3271

*∗* indicates significance.

**Table 4:** Acetabular failure descriptions.

**Failure Category**	**Description**
**1. Late acetabular loosening**	Thought to represent initial implant integration with subsequent failure of attachment. We divide late loosening into three subtypes for more detailed analysis

***1a. Debonding of the porous coating***	The main mode of interest in the current study. In these cases, initial fixation is achieved through bone ingrowth. At some point beyond 2 years, the well-fixed, porous layer from the component substrate “debonds”, causing subsequent implant loosening. The patients typically have excellent function prior to loosening with no change in acetabular component position on radiographs. They will then present with a 2-3-month prodrome of mild symptoms without radiographic findings before a sudden acute worsening of symptoms and an inability to bear weight on the affected leg. Loosening is then obvious radiographically with shifting of the acetabular component into a markedly steeper position. Intraoperative findings include a grossly loose acetabular component with a section of porous coating debonded from the implant substrate. This contiguous “sheet” of plasma spray coating, usually 20 to 30% of the total surface, is seen to be well fixed to the surrounding bone. The remainder of the porous coating remains attached to the implant and has no apparent bone ingrowth. There is a mild amount of metallosis and reactive fluid with no soft tissue mass. There is no fibrous pseudomembrane.

***2b. Loosening with intact porous coating***	Rarely occurs after 2 years without debonding. These cases may represent cases of initial fibrous ingrowth with radiographically stable implants for at least 2 years and subsequent loss of fibrous fixation. They present with late onset of chronic pain and late change in radiographic AIA. They have acceptable metal ion levels (<10 *µ*m/L) and small collections of serosanguinous fluid, with no significant metallosis, soft tissue masses, or muscle destruction. We exclude cases of AWRF (described below) from this group. Intraoperatively, the acetabular component is either grossly loose or easily detached with a tamp. There is a thin 2-3 mm fibrous membrane present, and the adjacent bone is typically quite hard.

***3c. Status of porous coating unknown***	Cases that have failed beyond 2 years and were revised elsewhere; therefore, we cannot categorize the mode of failure or status of the implant coating.

**2. Early acetabular loosening**	Occurs before 2 years and is thought to be due to failure of bone ingrowth into the acetabular component, which can present with an acute cup spinout or a gradual symptomatic shift. In both types, the component usually shifts into a more steeply inclined position. The porous coating is found intact, and in early cases, blood may be encountered where the soft tissue dissection from the original operation has not yet completely healed. In the later cases of gradual shift, there is usually a fibrous layer with serous fluid. Black Ti metallosis is rarely seen. We have not observed AWRF before 2 years.

**3.Adverse wear-related failure (AWRF)**	Caused by inadequate coverage arc [15, 31]. The relationship of AWRF to component bearing size, coverage arc, and implant AIA on standing radiographs was elucidated in our 2013 RAIL paper [31], which describes a safe zone for placing all cup sizes with a 99% confidence level. When these current study cases were performed, the importance of cup position was not yet appreciated. Therefore, a small percentage of AWRF occurred due to acetabular component malposition. These failures present with progressive chronic pain, usually after 5 years postoperatively. If routine blood metal ion testing at 2 years is undertaken, as recommended by DeSmet [15], these cases can be discovered before the patients become symptomatic and when soft tissue damage is minimal. Initially, radiographs are unremarkable except for unacceptably steep AIA (according to RAIL). Progressive femoral neck narrowing and lysis can occur in late cases. 3D studies show large fluid collections and soft tissue masses. Extensive grey tissue staining, milky tan fluid, and inflammatory soft tissue masses are encountered at revision. Usually, some metallic filled lysis is present. Bone loss is typically much less prominent than soft tissue inflammation. Component loosening is rare. We have never seen significant muscle destruction in HRA AWRF. In our experience, tissue inflammation in THA due to trunnion corrosion may be more severe with low ion levels and should be considered separately from HRA pure bearing wear. We emphasize that the tissue destruction seen in MoM THA cases cannot be considered equivalent to AWRF in HRA.

**Table 5:** Clinical and radiographic data.

Clinical and Radiographic Data			
*⁡⁡⁡*	Group 1	Group 2	

Variable	Corin	Biomet	P-value

n	**371**	**728**	--

Clinical Data			

Harris Hip Score (Post-Op)	93.6 ± 14.6	96.7 ± 7.6	0.0001*∗*

UCLA Score	6.6 ± 2.1	7.2 ± 2.0	0.0001*∗*

VAS Pain: Regular Day	0.6 ± 1.6	0.3 ± 0.9	0.001*∗*

VAS Pain: Worst Day	1.7 ± 2.6	1.4 ± 2.2	0.03*∗*

Mean Cobalt	2.6 ± 3.7	1.7 ± 1.8	0.0001*∗*

Mean Chromium	1.7 ± 2.3	1.1 ± 1.3	0.0001*∗*

Radiographic Data			

Acetabular Inclination Angle (°)	45.6 ± 7.2	45.1 ± 11.7	0.6504

Radiolucency	4 (0.01%)	0 (0.00%)	0.005*∗*

Osteolysis	3 (0.01%)	2 (0.00%)	0.200

*∗* indicates significance.

**Table 6:** Acetabular position data.

****	**Debonded Corin**	**ALL Corin**	**ALL Biomet**
*# Met RAIL*	4/13 (30.8%)	133/227 (58.6%)	265/506 (52.4%)

*Avg AIA*	48.0 ± 8.6	44.2 ± 7.3	45.1 ± 11.7

## Data Availability

The data used to support the findings of this study are available from the corresponding author upon request.

## Abbreviations

HRA: Hip resurfacing arthroplasty
THA: Total hip arthroplasty
MoM: Metal-on-metal
Ti: Titanium
Co: Cobalt
Cr: Chromium
PVD: Physical vapor deposition
HA: Hydroxyapatite
AWRF: Adverse wear-related failure
HHS: Harris hip score
UCLA: University of California Los Angeles
VAS: Visual analog scale
AIA: Acetabular inclination angle
KM: Kaplan-Meier
RAIL: Relative acetabular inclination limit
HIP: Hot isostatic pressing.
